# Use of Urethral Sound to Facilitate Locating Retrovesical Ureter for Politano-Leadbetter Pneumovesicoscopic Ureteral Reimplantation

**DOI:** 10.3389/fped.2022.834465

**Published:** 2022-03-03

**Authors:** Xiang Zhao, Qingqing Tian, Erhu Fang, Ning Li

**Affiliations:** Department of Pediatric Surgery, Tongji Hospital, Tongji Medical College, Huazhong University of Science and Technology, Wuhan, China

**Keywords:** Politano-Leadbetter, ureteral reimplantation, pneumovesicoscopic, urethral sound, vesicoureteral reflux, ureterovesical junction obstruction

## Abstract

**Background:**

Pneumovesicoscopic ureteral reimplantation (PVUR) has gained popularity due to its minimal invasiveness. However, most of the reported PVUR procedures were based on the Cohen technique. Only few studies reported their experience of PVUR using the Politano-Leadbetter technique (PVUR-PL). Here, we reported our experience of PVUR-PL using a novel technique to facilitate locating the retrovesical ureter during the procedure.

**Materials and Methods:**

The medical records of the patients who underwent PVUR-PL between January 2018 and December 2020 in our institution were retrospectively reviewed. The patients were classified into two groups: the modified group that accepted PVUR-PL using our novel technique (using urethral sound to facilitate identifying the retrovesical ureter) and the traditional group that accepted PVUR-PL not using the novel technique. Clinical data were collected retrospectively.

**Results:**

There were 22 patients who underwent PVUR-PL, with 13 in the traditional group and nine in the modified group. The mean operating time for unilateral cases in the modified group was significantly shorter than that in the traditional group (154.5 vs. 195.5 min, *p* < 0.001). For bilateral cases, the mean operating time was also significantly reduced (from 263.0 to 221.3 min, *p* = 0.022) in the modified group. There were no severe complications in each of the two groups. The peritoneum was perforated in one case from the traditional group, while no peritoneum perforation occurred in the modified group.

**Conclusion:**

The use of urethral sound to help to identify the retrovesical ureter during PVUR-PL is a safe and effective technique. This simple but effective technique could shorten the operating time of PVUR-PL and reduce the risk of peritoneum perforation.

## Contribution to the Field

Pneumovesicoscopic ureteral reimplantation (PVUR) has gained popularity due to its minimal invasiveness. However, only few studies reported their experience of PVUR using Politano-Leadbetter technique (PVUR-PL). Here, we reported our experience of PVUR-PL using a novel technique to facilitate locating the retrovesical ureter during the procedure. This technique just needs a urethral sound. During the operation, a urethral sound is inserted into the ureter through the urethra, and then the end of the sound is pushed forward to the location just beneath the new hiatus location of the bladder. By uplifting the end of the sound, the proximal ureter and the superjacent bladder wall will protrude into the bladder cavity, and then it will be very easy to open the new hiatus and identify the retrovesical ureter. This technique could shorten the operation time and reduces the risk of peritoneum perforation for PVUR-PL.

## Introduction

With the development of minimally invasive techniques, traditional open surgery is challenged by laparoscopic or endoscopic procedures. Ureteral reimplantation, which is usually recommended for patients with ureterovesical junction obstruction (UVJO) or severe vesicoureteral reflux (VUR), is also gradually replaced by laparoscopic procedures (1–4). Endoscopic injection for primary VUR or endoscopic balloon dilatation for UVJO also showed promising treatment outcomes (5–7). However, in some regions of the world, the lack of available injection materials hampers endoscopic injection for the management of primary VUR. Moreover, a small portion of VUR or UVJO patients initially treated with endoscopic procedures still need secondary ureteral reimplantation (5–7). Therefore, ureteral reimplantation is still the most popular treatment option for VUJO or VUR around the world (8).

Recent years, pneumovesicoscopic ureteral reimplantation (PVUR) has gained popularity due to its minimal invasiveness (1–4). However, most of the reported PVUR procedures were based on the Cohen technique, which passes the ureter through a cross-trigonal submucosal tunnel to the contralateral side (1–4). The main drawback is that it alters the natural course of the ureter and thus hampers future retrograde ureteral catherization or ureteroscope.

The Politano-Leadbetter ureteral reimplantation technique does not change the natural course of the ureter (9). It keeps the ureter in an anatomically natural course allowing future retrograde ureter catherization. However, the PVUR using Politano-Leadbetter technique (PVUR-PL) is more difficult to be performed than PVUR using Cohen technique due to its technical complexity. The main obstacle in PVUR-PL is how to establish the submucosal tunnel and pass the ureter through the tunnel. In 2015, Soh et al. (10) reported their experience of PVUR-PL, which requires an additional trocar through the urethra and the assistance of cystoscope in addition to laparoscope. In 2015 and 2016, Kim et al. (11) and Choi et al. (12), respectively, also reported their experience of PVUR-PL in which the bladder detrusor has to be split, just like the Lich-Gregoir technique. In 2019, Baek et al. (13) reported a simpler technique for PVUR-PL without the need of a cystoscope or cutting the detrusor. According to Baek's procedure, they open the proximal new hiatus first and then search for the retrovesical ureter through the newly opened window by pulling the end of the distal ureter (13). Although the procedure for PVUR-PL is simplified by Baek's technique, it is still difficult to identify the retrovesical ureter underneath the new hiatus.

In this article, we reported our experience of PVUR-PL and introduce a simple but effective novel technique to further simplify the procedure of PVUR-PL. This technique just needs a urethral sound. During the operation, a urethral sound [also called as urethral bougie, a metal probe often used for the management of urethral stricture (14, 15)] is inserted into the ureter through the urethra, and then the end of the sound is pushed forward to the location just beneath the new hiatus location of the bladder. By uplifting the end of the sound, the proximal ureter and the superjacent bladder wall will protrude into the bladder cavity, and then it will be very easy to open the new hiatus and identify the retrovesical ureter. We hypothesized that this technique may shorten the operation time and reduce the risk of peritoneum perforation for PVUR-PL.

## Materials and Methods

### Patients and Study Design

This study was approved by the Ethic Committee of Tongji Hospital (TJ-IRB20210933) and informed consent was exempted due to the retrospective design. The medical records of the patients who underwent PVUR-PL between January 2018 and December 2020 were retrospectively reviewed. Surgical indications for PVUR-PL include persistent UVJO, severe primary VUR (high-grade VUR or recurrent febrile urinary tract infections), and other types of vesicoureteral junction abnormality needed to be corrected by surgical intervention. All surgical operations were performed by the same surgeon (Li Ning). At the beginning, we performed PVUR-PL with the procedure similar to what Baek et al. reported (13). However, since the October of 2019, we introduced the novel technique applying urethral sound into the procedure of PVUR-PL. Thus, the patients were classified into two groups: the traditional group that accepted PVUR-PL with the procedure similar to Baek's technique and the modified group that accepted PVUR-PL using our novel technique. Data about patient age, gender, disease type, operating time, intraoperative events, postoperative complications, length of hospital stay, follow-up period, and treatment outcomes were collected and analyzed. The definition of the operating time was from the start of cystoscopy to the end of suturing the trocar ports. Patients with age less than 6 months were considered unsuitable candidates for PVUR-PL due to their small bladder capacity. Cases with a mega-ureter that needs to be tapered were also excluded from this study. Ultrasonography was performed 3, 6, and 12 months postoperatively. Voiding cystourethrography (VCUG) was performed about 6 months after surgery to determine VUR if necessary. Patents with a follow-up period less than 6 months were excluded.

The primary outcome of this study is the total operation time. Considering that the highlight of our novel technique is to help move the retrovesical ureter into the bladder, the time duration for pulling the ureter into the bladder is considered as one of the secondary outcomes. Surgical complications are also considered as a secondary outcome of this study.

### Surgical Technique

The procedure for PVUR-PL are as follows.

#### Port Placement

Patients are positioned supine with the legs separated. Transurethral cystoscopy is performed firstly and the bladder is fully filled with saline. Under the vision of a cystoscope, three stay sutures are punctured percutaneously into the bladder and then pulled up and tied, at the dome and each side of the bladder separately, in order to make the abdominal wall and the bladder wall closely attached. While the stay sutures are pulled up under the vision of a cystoscope, a 5-mm port is firstly inserted through the abdominal wall into the bladder cavity just nearby the middle stay suture inferiorly and fixed by the suture to avoid dislodgement. The second and third 5-mm ports are then inserted into each side of the bladder in the same manner and fixed by the corresponding stay suture.

**Figure 1:**
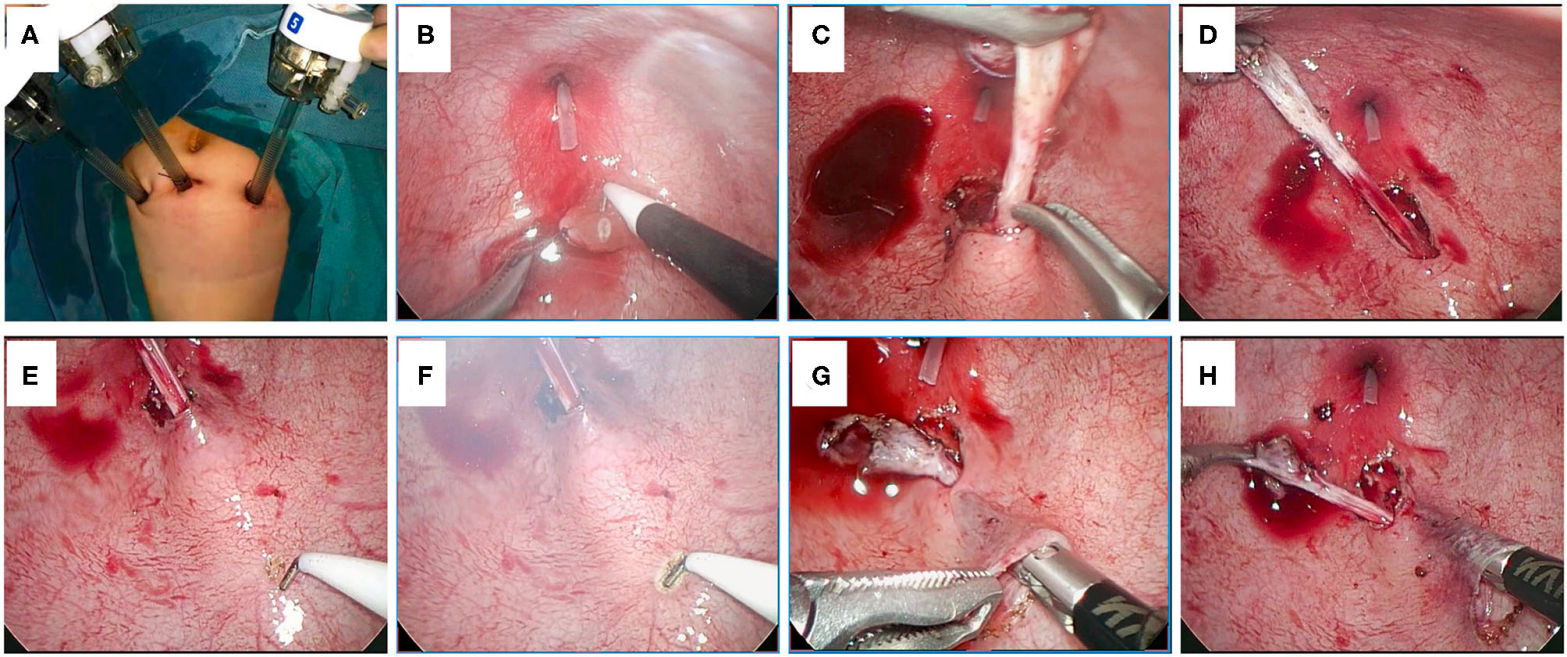
**(A)** The position of the trocars. **(B)** The ureteral orifice is sutured for pulling. **(C,D)** The distal ureter is mobilized to obtain an adequate length. **(E)** The distal ureter is gently pulled to show the course of the ureter. **(F)** The mucosa of the selected proximal new hiatus is dissected by an electric hook. **(G,H)** Establishment of the submucosal tunnel.

After the three ports are placed (Figure 1A), the cystoscope is removed and the saline in the bladder is evacuated. A urethral catheter is inserted into the bladder to occlude the urethra, and it could also function as a suction tube during the operation. The bladder is insufflated with CO_2_ at a pressure of 6 to 10 mmHg and a flow rate of 2 to 3 L/min.

#### Mobilization of the Distal Ureter

The middle port is used for laparoscope placement and the other two are used for manipulation. The affected ureteral orifice is firstly sutured using a 5-0 filament and then pulled up by gently drawing the suture (Figure 1B). Afterwards, a circumscribing incision is made around the ureteral orifice and the distal ureter is dissected circumferentially (Figure 1C). The distal ureter is mobilized until an adequate length was obtained (Figure 1D). To avoid damaging the blood supply of the ureter and injuring the vas deferens (in male patients), the operators should be careful when mobilizing the distal ureter. From our experience, at the beginning of dissecting, electronic cutting could be used; however, once the right plane (the plane between ureteral adventitia and bladder detrusor muscle) was entered, blunt dissection should be given priority to, whereas electrocoagulation could be used for hemostasis in case of bleeding.

#### Establishment of the Submucosal Tunnel

After an adequate length of the distal ureter is obtained, the distal ureter is gently pulled and the course of the ureter behind the bladder wall could be manifested (Figure 1E). Then, the location of the proximal new hiatus (superior to the original hiatus usually with a distance five times the ureteral diameter) could be selected along the raised ureter course and the mucosa at this location is dissected by an electric hook (Figure 1F). Afterwards, a submucosal tunnel is created between the proximal new hiatus and the original hiatus. The dissection of the mucosa layer and the detrusor layer starts at the proximal hiatus using laparoscopic forceps through the ipsilateral port (Figures 1G,H). Another pair of forceps could help to elevate the mucosa layer nearby the new hiatus at the beginning of dissection (Figure 1G).

#### Passing the Distal Ureter Through the Submucosal Tunnel

In the traditional group, the proximal new hiatus of the bladder wall is opened and the retrovesical ureter is searched through the newly opened window by pulling the end of the distal ureter per Baek's technique (13). When the retrovesical distal ureter is found, it is further mobilized and then pulled into the bladder.

**Figure 2:**
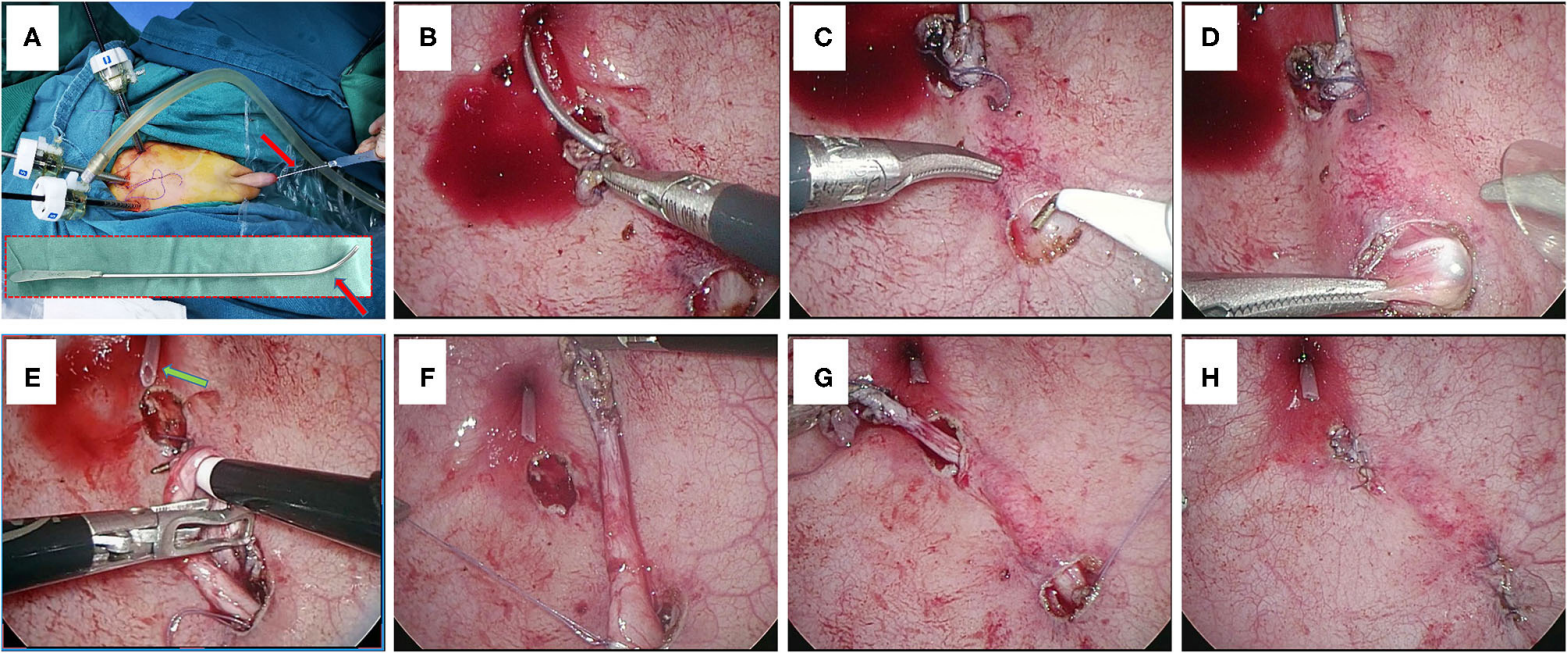
**(A)** Placement of a urethral sound through the urethra. Red arrows indicate the urethral sound. **(B)** The urethra sound is inserted into the ureter. **(C)** The end of the urethral sound is pushed forward to reach the location of the new hiatus. **(D)** The retrovesical ureter is uplifted by the end of the urethral sound. **(E)** The distal ureter is further mobilized through the new hiatus. Green arrow indicates the urethral catheter. **(F)** The distal ureter is pulled into the bladder through the new hiatus. **(G)** The distal ureter is moved through the submucosal tunnel. **(H)** The ureteral orifice is anastomosed to the original hiatus.

In the modified group, the urethral catheter that serves as a suction tube in the urethra was removed, and a urethral sound (Figure 2A, red arrow) is placed into the bladder through the urethra and then inserted into the ureter through the orifice of the ureter (Figures 2A,B). The end of the urethral sound in the ureter should reach the location just beneath the new hiatus location of the bladder (Figure 2C). By uplifting the end of the urethral sound, the retrovesical ureter as well as the superjacent bladder wall (the location of the proximal new hiatus) will protrude into the bladder cavity (Figure 2D). This technique, which is the main improvement in our procedure, would facilitate the finding of the retrovesical ureter when opening the new hiatus on the bladder wall. The detrusor muscle in the location of the protruding new hiatus is dissected and the retrovesical ureter will be very easily found (Figure 2D). Once the correct layer was entered and the retrovesical ureter was found, the urethral sound was removed and the urethral catheter was inserted into the urethra again for suction again (Figure 2E, green arrow). The ureter will then be further mobilized through the proximal new hiatus until the distal ureter could be pulled into the bladder (Figures 2E,F). Afterwards, the distal ureter is moved from the proximal hiatus to the original distal hiatus through the submucosal tunnel (Figure 2G).

#### Anastomosis of the Ureteral Orifice

The proximal ureter nearby the proximal hiatus is fixed with the adjacent detrusor muscle by a couple of 4-0 interrupted absorbable sutures. The mucosal defect at the proximal hiatus is closed by 5-0 absorbable sutures. The detrusor defect in the distal original hiatus is closed by 4-0 absorbable sutures. Pathological and redundant terminal ureter is excised and then the ureteral orifice is anastomosed to the original hiatus with four or five 5-0 absorbable sutures. The first two anastomotic sutures should pass through both the bladder mucosa and detrusor muscle to acquire a feasible level of steadiness. The final course of the ureter should be in a physiologically and anatomically natural trend (Figure 2H).

### Statistical Analysis

Categorical variables in different groups were compared by Fisher's exact test. Continuous variables in different groups were compared by Student's *t*-test. The statistical analyses were performed using IBM SPSS software (version 18) and R software (version 4.1.0); a *p*-value of <0.05 was considered as statistically significant.

## Results

There were a total of 22 patients who underwent PVUR-PL (Table 1), and no patient was lost to follow-up. Thirteen patients underwent traditional type PVUR-PL. Nine patients underwent modified-type PVUR-PL using our novel technique. In the traditional PVUR-PL group, 10 patients were with VUR (seven unilateral and three bilateral) and the other three were with UVJO. In the modified PVUR-PL group, seven patients were with VUR (four unilateral and three bilateral) and the other two were with UVJO. One UVJO patient who underwent modified PVUR-PL was accompanied by ureterocele and ureteral calculi in the distal ureteral.

**Table 1:** Clinical characteristics of the patients in the two groups.

**Patients**	**Traditional group**	**Modified group**	***p*-value**
Case numbers	13	9	–
Gender			0.99
Male	6	4	
Female	7	5	
Age (years)	Median: 2.5 IQR: 0.9–6.0	Median: 4.3 IQR: 1.8–5.0	0.99
VUR			0.99
Left	5	3	
Right	2	1	
Bilateral	3	3	
VUR grade			0.99
Grade III	5	3	
Grade IV	7	6	
Grade V	1	1	
UVJO			0.99
Left	2	1	
Right	1	1	

*IQR, interquartile range; VUR, vesicoureteral reflux; UVJO, ureterovesical junction obstruction*.

For unilateral cases, the mean operating time was 195.5 [median: 189.5, interquartile range (IQR): 184.0–210.75] min in the traditional group, while it was 154.5 (median: 154.5, IQR: 148.75–161.0) min in the modified group (*p* < 0.001, Table 2). After excluding the first five cases in the traditional groups, the mean operating time was 179.0 (median: 183.0, IQR: 175.0–188.0) min, which was still longer than that in the modified group (*p* = 0.004, Table 2). For bilateral cases, the mean operating time was 263.0 (median: 263.0, IQR: 255.0–271.0) min in the traditional group, while it reduced to 221.3 (median: 221.0, IQR: 215.5–227.0) min in the modified group (*p* = 0.022, Table 2).

**Table 2:** Treatment outcomes for each group.

	**Traditional group (*n* = 13)**	**Modified group (*n* = 9)**	***p*-value**
Operation time (min) for unilateral cases	Median: 189.5 IQR: 184.0–210.75	Median: 154.5 IQR: 148.75–161.0	<0.001
Operation time (min) for unilateral cases (after excluding the first 5 cases	Median: 183.0 IQR: 175.0–188.0	Median: 154.5 IQR: 148.75–161.0	0.004
Operation time (min) for bilateral cases	Median: 263.0 IQR: 255.0–271.0	Median: 221.0 IQR: 215.5–227.0	0.022
Time (min) for pulling the ureter into the bladder	Median: 44.0 IQR: 41.0–46.0	Median: 26.0 IQR: 22.0–28.0	<0.001
Peritoneum perforation	1	0	0.99
Febrile UTI	1	0	0.99

*IQR, interquartile range; SD, standard deviation; UTI, urinary tract infection*.

**Figure 3:**
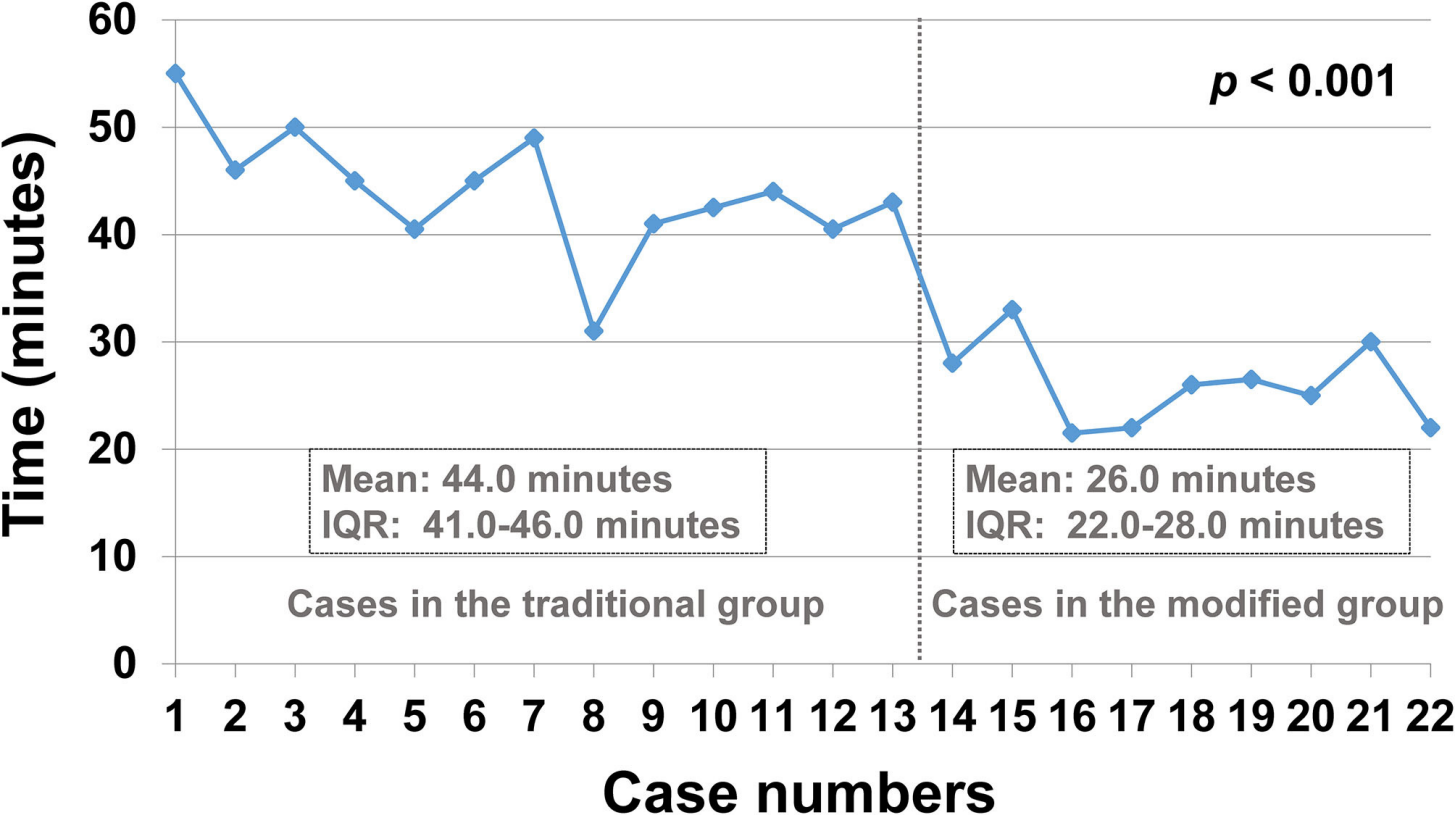
The time used for pulling the ureter into the bladder for each case in chronological order. IQR, interquartile range.

Considering that the highlight of our novel technique is to help find the retrovesical distal ureter and move it into the bladder, we also calculated the time period of this step for each of the two groups according to the operation videos. For the traditional group, the time period was defined from opening the proximal hiatus to finishing pulling the distal ureter into the bladder. For the modified group, the time duration was from placing the urethral sound into the ureter to finishing pulling the distal ureter into the bladder. Figure 3 shows the time duration of this step for each case in chronological order. The mean time for this step in the traditional group was 44.0 (median: 44.0, IQR: 41.0–46.0) min, while it reduced to 26.0 (median: 26.0, IQR: 22.0–28.0) min in the modified group (*p* < 0.001, Table 2).

There were no severe complications during the operation in each of the two groups. The peritoneum was perforated when searching for the retrovesical ureter through the proximal hiatus in one case from the traditional group. However, the operation was successfully continued by placing a Veress needle to evacuate the gas in the abdomen.

One unilateral VUR patient in the traditional group underwent febrile urinary tract infection (UTI) 4 days postoperatively. This patient was treated with intravenous antibiotics and recovered well. No more febrile UTI was occurred for this patient during the follow-up period and no VUR was detected by VCUG 6 months later postoperatively.

None of the other patients underwent febrile UTI during the follow-up period. Contrast-enhanced voiding ultrasonography (CVUS) was performed in three of the seven patients in the modified group and in five of the 10 patients in the traditional group, and no VUR was detected. The VCUG or CVUS was not performed in the other patients since no febrile UTI occurred during the follow-up period.

## Discussion

According to the literature, there are mainly three types of minimally invasive ureteral reimplantation: the Lich-Gregoir technique, the Cohen technique, and the Politano-Leadbetter technique (1–4, 10, 12, 13). The extravesical Lich-Gregoir laparoscopic ureteral reimplantation is usually recommended to patients with VUR (10, 12). The advantage of the laparoscopic Lich-Gregoir technique is that it is able to keep an orthotopic ureteral location that allows for future retrograde ureteral catheterization, while its disadvantage is that it disturbs the abdominal cavity and dissects the bladder detrusor, raising the concern of potential injury to pelvic nerves and the resultant bladder emptying neurogenicity (3, 4). The PVUR using the Cohen technique could be used for both UVJO and VUR patients. It is a procedure without the above-mentioned disadvantages of the Lich-Gregoir technique, but it occludes future retrograde ureteral catheterization because of the cross-trigonal change of the ureteral course (1, 2, 10).

The Politano-Leadbetter ureteral reimplantation technique overcomes the above-mentioned limitations of the Cohen technique and the Lich-Gregoir technique. Instead of creating an angled cross-trigonal submucosal tunnel, the Politano-Leadbetter technique establishes the submucosal tunnel in a naturally straight trend, allowing future retrograde ureter catherization (9). The open Politano-Leadbetter technique also does not have to split the detrusor muscle like what the Lich-Gregoir technique does (9). However, the Politano-Leadbetter technique is more complex than the Cohen technique and the Lich-Gregoir technique. It is very challenging to perform PVUR using the Politano-Leadbetter technique due to the technical complexity. The main challenge of PVUR-PL is creating the submucosal tunnel and passing the ureter through the tunnel. Until now, few surgeons reported their experience of PVUR using the Politano-Leadbetter technique (10–13, 16, 17). In order to facilitate the creation of the submucosal tunnel and move the ureter through the tunnel, Soh et al. (10) inserted an additional trocar through the urethra and utilized a cystoscope in addition to a laparoscope to facilitate the manipulation, whereas Choi et al. (12) and Kim et al. (11) split the bladder detrusor in their PVUR-PL procedure somewhat like what the Lich-Gregoir technique does.

In 2019, Baek et al. (13) reported a simpler technique for PVUR-PL without the need of a cystoscope or cutting the detrusor. Their technique is as follows: after the distal ureter is mobilized, they opened the proximal new hiatus and then searched for the retrovesical ureter through the newly opened window by pulling the end of the distal ureter; when the retrovesical ureter was found, they pulled it into the bladder cavity through the window and then passed it through the submucosal tunnel like the procedure in the open Politano-Leadbetter technique. In 2021, Beytullah et al. (17) also reported their experience of PVUR-PL with the procedure similar to which Baek et al. reported. Although the procedure for PVUR-PL is simplified by Baek's technique, it is still difficult to identify the retrovesical ureter underneath the new hiatus. As they have mentioned, it took a long time to find the ureter through the newly opened hiatus in the early stage of surgical experience. Although the time for the procedure reduced as the number of operations increased, it is still challenging for beginners. Another drawback of Baek's technique is the increasing risk of peritoneal perforation with the longer time of searching for the retrovesical ureter through the newly opened hiatus.

From 2018, we started to perform PVUR-PL with the procedure similar to which Baek et al. reported. We also attempted to find the retrovesical ureter from the cephalad newly opened hiatus. There were mainly two challenging steps during the process. The first is to dissect the bladder wall just into the correct layer (the plane between the detrusor muscle and the ureter adventitia) and not perforate the peritoneum. The second is to find the retrovesical ureter from the newly opened hiatus and pull it into the bladder cavity. Indeed, if the correct plane was entered, the search for the retrovesical ureter would be much easier by pulling the tail of the ureter. However, it is still somewhat challenging for each of these two steps, especially in the early stage of experience.

In order to facilitate the identification of the retrovesical ureter from the newly opened hiatus, we attempted to use a ureteral sound as a guide by inserting it to the ureter. The end of the ureteral sound was pushed forward into the ureter until it reached the location just beneath the new hiatus location of the bladder wall. By uplifting the end of the urethral sound, the retrovesical ureter as well as the superjacent bladder wall (the location of the proximal new hiatus) will protrude into the bladder cavity. This technique, although simple but effective, make it much easier to dissect the bladder wall into the correct plane and to find the retrovesical ureter through the newly opened window. Since there is no complicated manipulation, this technique can be done with little effort even for beginners.

In our experience, the time for identifying the retrovesical ureter and pulling it into the bladder was significantly reduced (from an average of 44.0 to 26.0 min). After applying this novel technique, the total operation time was reduced from an average of 179.0 to 154.5 min for unilateral PVUR-PL and was reduced from an average of 263.0 to 221.3 min for bilateral PVUR-PL. Furthermore, no peritoneum perforation occurred after using this novel technique.

In conclusion, the use of urethral sound to help to identify the retrovesical ureter during PVUR-PL is a safe and effective technique. This simple but effective technique could shorten the operating time of PVUR-PL and reduce the risk of peritoneum perforation. It is very easy to manipulate, even by beginners, and thus makes the procedure of PVUR-PL less complicated. We believe that this technique could help more surgeons to perform PVUR using the Politano-Leadbetter technique.

## Data Availability Statement

The original contributions presented in the study are included in the article/supplementary material, further inquiries can be directed to the corresponding author/s.

## Ethics Statement

The studies involving human participants were reviewed and approved by the Medical Ethics Committee of Tongji Hospital of Tongji Medical College of Huazhong University of Science and Technology. Written informed consent from the participants' legal guardian/next of kin was not required to participate in this study in accordance with the national legislation and the institutional requirements.

## Author Contributions

XZ: original manuscript writing and data analysis. NL: supervising, manuscript revising, and study design. EF, QT, and XZ: data collection. All authors read and approved the final manuscript.

## Conflict of Interest

The authors declare that the research was conducted in the absence of any commercial or financial relationships that could be construed as a potential conflict of interest.

## Publisher's Note

All claims expressed in this article are solely those of the authors and do not necessarily represent those of their affiliated organizations, or those of the publisher, the editors and the reviewers. Any product that may be evaluated in this article, or claim that may be made by its manufacturer, is not guaranteed or endorsed by the publisher.
